# Strategies Employed by Nursing Managers Within a Transformational Approach: A Qualitative Study

**DOI:** 10.1155/nrp/9309685

**Published:** 2025-02-27

**Authors:** Gholamhossein Mahmoudirad, Ayob Akbari

**Affiliations:** Nursing and Midwifery School, Birjand University of Medical Sciences, Birjand, Iran

**Keywords:** nurse Administrators, nursing, qualitative research

## Abstract

**Background:** The roles of nursing managers are diverse and demand high proficiency, with transformational approach recognized as a key competency for achieving organizational goals. A transformational approach is identified as essential in addressing the unique challenges of healthcare management, particularly in nursing, by enhancing self-efficacy and fostering trust among team members.

**Design:** This study employed a conventional qualitative content analysis design.

**Methods:** Purposive sampling was used to recruit 23 nurse managers from hospital settings between April 2022 and August 2023. Data were collected through semistructured interviews, which were audiorecorded with participants' consent, transcribed verbatim into Word documents, and imported into MAXQDA software (Version 2020) for systematic organization and analysis. The data were analyzed using Graneheim and Lundman's (2020) qualitative content analysis method, while trustworthiness was ensured based on the criteria proposed by Kyngas, Kaariainen, and Elo (2020).

**Results:** Eight subcategories and three final categories were identified as key strategies used by nursing managers in a transformational approach. The findings revealed that nursing managers employ strategies such as drawing the path of transformation, fostering a transformation-based culture, and facilitating transformational change within their practices.

**Conclusion:** This study emphasizes the transformational strategies employed by nursing managers, which involve establishing clear pathways for transformation, fostering a culture of change, and acting as facilitators of the transformation process. It is recommended that nursing managers consistently implement and refine these strategies to promote innovation, adaptability, and a culture centered around change and transformation. Through these efforts, nursing managers can significantly enhance organizational effectiveness, improve patient outcomes, and drive substantial advancements in nursing practice.

## 1. Background

As direct care providers, nurses and nursing managers play a pivotal role in driving changes within the healthcare system [1, 2]. Nursing management, positioned at the forefront of this transformation, requires active and committed managers to successfully implement workplace changes [3, 4]. The roles and responsibilities of nursing managers in hospitals vary significantly across countries, reflecting differences in healthcare systems and organizational structures. These roles are multifaceted and demand a high level of proficiency and expertise [5, 6]. Nursing managers must leverage their skills to develop essential competencies aimed at achieving organizational objectives. Moreover, leadership in nursing is widely recognized as a critical competency for attaining these goals [7, 8].

In hospital settings, there is a pressing need for leaders and managers to adopt a transformational approach tailored to the unique demands of healthcare management, particularly in nursing. Nurse leaders, in this context, play a vital role by providing education, supervision, and support to their teams [9, 10]. The transformational approach is recognized as an effective strategy for leaders and managers to address workplace challenges, making it essential to identify actionable strategies that facilitate its implementation [11]. The integration of leadership strategies is a critical component of effective care administration and an indispensable managerial skill. In the current healthcare climate, these strategies are particularly significant for improving nurses' performance and fostering adaptability within nursing practice [11].

Adopting a transformational leadership style that enhances the self-efficacy of change recipients is essential for fostering positive attitudes and behaviors during organizational changes. This approach becomes particularly critical when managing extensive changes [12]. Implementing a transformational approach in the workplace builds trust among team members, thereby improving overall effectiveness [13]. As highlighted by Wu, this leadership style can help mitigate nurses' burnout and, by fostering a positive spiritual climate, enhance the perceived significance of their work. These factors are pivotal in improving nurse retention rates [14].

Leadership characteristics significantly shape leadership styles, which impact organizational outcomes and strategic direction. In Asia, unique sociocultural factors, including philosophies and values, influence the development of leadership styles distinct from those in other regions. However, there is limited research exploring how these characteristics manifest within Asia's specific sociocultural context [15–17]. The application of transformational leadership across countries, especially within diverse sociocultural contexts like Asia, provides critical insights into the unique strategies and challenges faced by nursing managers in various healthcare settings. Key leadership practices, such as communication, trust building, and emotional support, are crucial for enhancing nurse retention and cultivating positive work environments. However, there remains a gap in understanding how these strategies are applied in practice, particularly in healthcare systems where variations in resources, organizational priorities, and cultural norms influence their implementation.

A comparative evaluation of these strategies across different settings can help refine leadership approaches that are both universally effective and contextually appropriate. A review of the existing literature identified a critical gap concerning the strategies employed by nursing managers within a transformational approach. While the impact of transformational leadership on organizational outcomes is well-documented, there is insufficient exploration of the practical strategies managers use to implement this leadership style. To address this gap, the present study adopted a qualitative research methodology, enabling an in-depth understanding of these strategies.

A qualitative approach allows participants to articulate their thoughts, feelings, and experiences in their own words, providing insights into the how, why, and what of their actions during specific events or periods of interest. Considering nursing managers operate at the intersection of patient care and organizational leadership, their perspectives are vital to comprehending how transformational strategies are developed and applied in practice.

In light of this, the study was guided by the research question: “What strategies are adopted by nursing managers in a transformational approach?” The objective of this study was to explore and elucidate these strategies to inform and enhance nursing practice.

## 2. Materials and Methods

### 2.1. Study Design and Participant

The study employs conventional qualitative content analysis to explore the strategies employed by nursing managers within a transformational approach. The choice of this method was guided by its ability to systematically interpret and analyze open-ended data, making it particularly valuable for capturing the nuanced experiences and strategies of nursing managers [18]. Conventional content analysis is well-suited for studies aiming to derive themes directly from the data without imposing preconceived categories, allowing for a comprehensive understanding of the phenomena under investigation [19].

Between April 2022 and August 2023, nursing managers from educational hospitals in Birjand, Iran, were purposively sampled for the study. The sample included head nurses, supervisors, and matrons, selected to ensure the maximum variation in terms of age, gender, and work experience. Eligible participants were required to have at least 5 years of work experience, with a minimum of 2 years in a management role, and must have consented to participate in the study. Those who declined to participate in interviews or were unwilling to share personal experiences were excluded from the study.

### 2.2. Data Collection

Data collection was carried out through in-depth, semistructured face-to-face interviews conducted by the corresponding author, Mr. A.A., Ph.D. (Assistant Professor of Nursing). These interviews took place in a quiet, private setting, after working hours, and lasted approximately 50 to 110 min, with an average duration of 80 min. Both the first and corresponding authors are experienced qualitative researchers, with multiple published articles across various academic journals. The participants demographic data, including age, gender, education level, current position, and work experience in ward management, were recorded.

Before initiating the interviews, the researcher established a professional and effective relationship with participants by explaining the research objectives and interview process to ensure their confidence and readiness. Participants had sufficient prior knowledge about the researchers, which included the study's objectives, reasons for conducting the research, and the researchers' personal interest in the topic. Necessary explanations were provided to build trust and motivate participation. The researcher demonstrated neutrality and effective communication skills across interviews.

The interviews began with an introductory question, inviting participants to share their experiences of a typical working day. Subsequently, probing questions were asked to gain a more detailed and nuanced understanding of their experiences. Examples of these probing questions are provided in Table 1. Data collection was concluded when all categories had been thoroughly explored, and no new data emerged.

With the participants' consent, all interviews were audiorecorded, transcribed verbatim into Word documents and subsequently loaded into MAXQDA software (Version 2020) for structured management and analysis of the qualitative data. A total of 26 interviews were conducted with 23 participants. Three participants were interviewed twice, and none of the participants withdrew from the study either during or after the interview process.

### 2.3. Data Analysis

The data were analyzed using the conventional qualitative content analysis method, which is a systematic approach designed for examining qualitative data. This method has evolved from its initial, straightforward form into a more interpretive approach. It is important to note that all analytical processes, regardless of the methodology employed, involve varying degrees of abstraction and interpretation [20].

In line with the guidelines established by Graneheim and Lundman for conventional content analysis, a five-step process was followed to analyze the interviews: (i) the recorded interviews were transcribed, (ii) the transcripts were thoroughly reviewed, with repeated listening, to identify meaningful segments, (iii) these meaningful units served as the basis for generating initial codes, (iv) similar codes were grouped according to their conceptual similarities, and (v) this process was applied to all units of analysis until categories and themes were identified [20, 21].

### 2.4. Ethics

This study received approval from the Ethical Committee of Birjand University of Medical Sciences. Participation was entirely voluntary. Participants were informed that their interviews would be recorded and assured that the data collected would remain confidential. Informed consent was obtained from all participants, who were also assured that they could withdraw from the study at any time without consequence. Additionally, participants were guaranteed that all information provided would be kept confidential.

### 2.5. Rigor

To ensure the trustworthiness of the data, this study employed the five criteria outlined by Kyngas, Kaariainen, and Elo [22], namely, credibility, dependability, conformability, authenticity, and transferability.

To enhance credibility, data collection was conducted over a period of approximately 8 months, ensuring the inclusion of participants from diverse age groups and genders. Dependability was achieved by subjecting the interview process, coding, and analysis to peer checking and review by external experts. Conformability was ensured through long-term engagement, multiple readings of the interviews, member checks, and participant verification of the codes.

Authenticity was guaranteed by systematically incorporating citations throughout the text, with each identified category containing at least one relevant citation. Additionally, quotations from various participants were included to reflect a range of perspectives. For participant identification, specific codes were used, such as “MS15,” where “M” denotes the male, “S” represents the supervisor, and “15” indicates the 15 years of experience.

To enhance transferability, the study employed maximum diversity sampling and member checks. Detailed descriptions of participant characteristics and the study context were provided, allowing readers to assess the applicability of the findings to their own environments. The transcripts were returned to the participants for feedback, and they confirmed the accuracy of the data; in addition, the study's results were presented with three nursing managers (head nurse, supervisor, and hospital matron) outside the study, who affirmed that the findings closely aligned with their own experiences.

## 3. Results

In this study, the number of male nursing managers participating (*n* = 12, 52%) was one more than the number of females (*n* = 11, 48%), with the majority holding a master's degree (*n* = 13, 56.5%). Additionally, eleven participants were head nurses (47.8%). The average professional experience of the participants was 18 (4.123) years, with a mean of 11 (5.47) years of management experience (Table 2).

**Figure 1:**
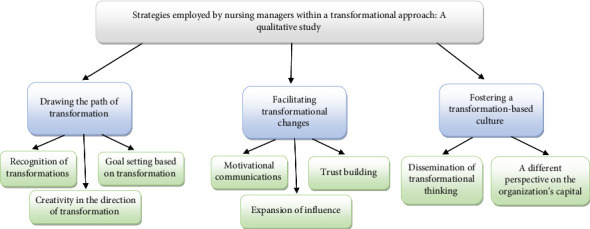
Coding tree.

Upon analyzing the data, 589 open codes, eight subcategories, and three final categories were created. The three main categories include drawing the path of transformation, fostering a transformation-based culture, and facilitating transformational changes (Figure 1).

(Participant identification guidance: M (Male), F (Female), H (Head Nurse), S (Supervisor), Ma (Matron))

### 3.1. Drawing the Path of Transformation

Drawing the transformation path is related to determining the direction and goals of the organization's transformation. Designing a transformational path means determining a plan and strategy for the professional growth and development of nursing managers, helping them improve their managerial and leadership competencies toward transformation.

Nursing managers consider three necessary strategies in the direction of transformation, which include the recognition of transformations, goal setting based on transformation and creativity in the path of transformation.

#### 3.1.1. Recognition of Transformations

After analyzing the experiences of nursing managers, we consider the first step in drawing the path of transformation to be the recognition of transformations. The recognition of transformations means increasing and expanding the knowledge, information, experience, and cognitive capabilities of nursing managers, helping them identify clinical and managerial transformations and provide appropriate planning to advance the transformation and progress of the organization.

Nursing managers consider constant thinking about transformations, synergy of knowledge and experience to get familiar with transformations, and gain recognition by going through management steps and establishing extensive communication as the most important strategies of transformation

*“I consider the fact that I think about changes and transformations in my field and follow transformations as my strength. I consider thinking about transformations, especially systemic thinking, to be important and the origin of transformations throughout the whole process. In addition to myself, I always think about making other nurses interested in thinking about transformations in their field” (MH17).*

Synergy of knowledge and experience is closely related to transformation in the field of nursing. Knowledge and experience should be developed simultaneously, combined, and clinically oriented so that nursing managers can achieve recovery and transformation.

*“We hold training workshops every week to cover educational needs. Personally, I keep my knowledge up-to-date by studying and researching, and I try to get the latest information about nursing transformations by searching a lot, and add to my own knowledge and experience”* (FH15).

Going through managerial steps in nursing, including head nurse, supervisor, and matron, gives managers the opportunity to gain knowledge and experience by facing managerial challenges and responsibilities, enabling them to understand and manage changes and transformations in the best way.

*“In the field of nursing, I establish communication and interaction at different levels and get a lot of information…. I was a nurse in the emergency department, I worked shifts with the same nurses, and then I became the head nurse, and now I am a supervisor…. I want to say that I have enough knowledge and understanding of emergency department problems, and I can recognize and plan transformations accordingly” (MH18).*

#### 3.1.2. Goal Setting Based on Transformation

When a nurse manager decides to achieve transformation and improve their processes and services, they must be able to design transformational goals.

Nursing managers consider the identification of transformational goals and mastery of goals, a holistic view on goals–values–transformations, and comprehensive monitoring and analysis of goals important for this stage. An interesting point deduced from the experiences of the participants was that they named goals, values, and transformations as three sides of a triangle.

*“I consider the goals of values and developments together. After I put system values, professional values, and social values together, I write my goals…. For example, I want to expand ethics in my field; before that, I identify moral values, such as good manners with patients, honesty with the personnel, etc., and based on these values, I set a goal that, in the end, I can create a transformation in ethics with proper planning” (MMa24).*

Also, a holistic view of goals-values-transformations helps nursing managers examine the transformations more comprehensively and from different aspects.

The last step is to analyze and monitor the goals. Analyzing and monitoring goals helps managers make the best decisions to achieve their goals and transformations with a clear vision and sufficient information. Without analyzing and monitoring goals, a correct and comprehensive understanding of changes and transformations will not be possible, and the change process will probably fail.

*“An example of a goal I set is to improve care quality and reduce complaints within the next year” (FS18)…. “After designing my goals based on values and mastering my goals, I analyze day by day whether it is suitable for my work field or not. And if I feel that they may not be applicable here, I change the goals according to the values of my field” (FH19).*

#### 3.1.3. Creativity in the Direction of Transformation

Creativity can play an important role in drawing the path of transformation. Drawing the path of transformation requires innovative approaches and ideation to determine appropriate and creative transformational goals and strategies. Creativity in the path of transformation in the experiences of nursing managers is associated with creativity in designing transformations in the heart of goals and values, creative thinking in discovering and rooting problems and creating a creative atmosphere and stimulating thinking.

When changes are placed at the heart of goals and values, managers can shape their decisions and efforts based on the values and principles that are important to them and in line with the nursing practice.

*“One of the things that helped me a lot to write the goals of the institution is that I try new ideas and ways in my goals, I connect the transformations to the values and goals” (MMa24, MS17).*

Some managers say: *“Our systems are not creative, and creative staff are not seen in the system. They believe that for innovation in nursing, creative staff should be identified, and environment for their growth should be provided”(FMa19), (FS22).*

*“Creative and thoughtful staff, unfortunately, receive less attention in the system. We formed a team and invited creative staff from different levels of nursing and other hospital staff to sit together and discuss…to Help identify and RCA the problems” (MS13).*

*“In order to ensure team success, I explore new ways and utilize innovative ideas across all aspects of our work (MH25)”*

### 3.2. Fostering a Transformation-Based Culture

In this study, nursing managers have been observed to employ varied strategies to foster a transformation-based culture. The participants stated that they could effectively foster the creation of a transformative culture in nursing by using the strategies such as dissemination of transformative thinking and a different perspective on the organization's capital.

#### 3.2.1. Dissemination of Transformational Thinking

Nursing managers who are interested in transformational thinking are always in search of knowledge and better understanding of issues. They constantly have more questions in their minds and seek answers, analyzing and examining issues more deeply to find new and better solutions.

According to nursing managers' experiences, the establishment of a transformation-based approach within an organization requires the widespread adoption of transformational thinking. To achieve this goal, they have recognized several strategies, including having a transformational mindset, knowing and teaching transformational thinking, introducing the values of transformational thinking, and creating a transformational organization.

The development of a transformational mindset means having more interaction with nursing transformations and a greater perception of what the transformation entails. It means that the nursing manager's attitude should be focused on perceiving more clinical changes and value-oriented transformations.

*“I am always thinking about improvement and progress in the organization. I also use the thoughts and ideas of staff to improve processes and care. I always review in my mind how I can improve in providing services?” (MMa11).*

Transformational thinking education helps nurses and other members of the treatment team to learn the necessary skills for creative thinking, innovation, risk acceptance, and change management in their work environment.

*“My goal is for the personnel to be familiar with these transformations and to be able to teach them to others. In this way, I can spread transformational thinking in the organization” (MS12).*

By introducing the values of transformational thinking and familiarizing the organization's personnel with them, awareness of the importance and need for transformational thinking in the organization and society increases synergistically. Different people get to know these values and use them, serving as role models for others, leading to the spread of transformational thinking among staff. This public awareness can encourage all staff to choose a transformational approach in solving the problems and changes ahead.

*“This transformational thinking is excellent. In the meetings where we think together, I talk more about the benefits of this type of thinking. The personnel also become interested when they see that problems are solved with creative thinking… (FH17).”*

A transformational organization continuously encourages and implements changes and transformations. These organizations seek to discover and create innovative solutions to face challenges, improve performance, and increase productivity.

*“With the cooperation of all the managers, we try to spread transformational thinking. The personnel learn to think about the changes and transformations in their field, so that eventually the organization is filled with transformational thoughts, this is very good. When did the organization learn how to create changes? Then I reached my goal” (MMa24).*

#### 3.2.2. A Different Perspective on the Organization's Capital

A different look at the capital of the entire organization means seeing organizational capital with a new and innovative approach. Instead of being limited to the usual and common views about organizational capital, such as financial resources, technical equipment, human resources, and substructures, this new view emphasizes more on the less common or intangible aspects of this capital.

Nursing managers have a different view of the organization's capital, finding ways to improve relationships by opening communication, valuing the capital of the organization, directing the nursing resources toward nursing benefits, and properly directing human capital with appropriate selection and appointment.

Organizational and transorganizational communication can help achieve common goals between organizations and allow everyone to work together. By creating this type of communication, it becomes possible to achieve better and more effective results in different fields of nursing.

*“We have opened the communication channels of the organization and we welcome all comments, we will try to listen more…. I establish communication with employees and other organizations based on mutual respect, and use the potential of each employee for improvement and transformations” (FMa19).*

Valuing the capital of the organization means giving importance and paying attention to the various resources and activities available to the organization. First, different capital of the organization should be identified, evaluated, and then managed.

*“I value personnel and other capital of the organization from the lowest to the highest level and rely on the abilities of each one of them” (MH18).*

In the process of directing nursing resources to nursing benefits, the exploitation and optimization of the existing resources of the organization are done in such a way that they create value and quality, resulting in the best possible results and performance.

*“Many managers do not pay attention to this issue and waste the organization's resources. But we have identified the weak point here and provided them with the resources they need. This justice of allocating resources has led to the quality of healthcare and client satisfaction” (FS23).*

Among the things raised about meritorious selection, it is possible to mention having the right criteria for choosing staff with clinical competence, choosing managers based on records and experience, and selecting managers based on merit rather than relationships. Among the things raised about competent appointments, it is not choosing people based on relationships, considering the criteria related to giving a post to the personnel, and not hiring commissioned and partisan staff.

*“My most important capital is human resources. I believe that the right people should be in the right places, and then move towards transformation, so I will observe justice in two cases. One is justice in selection, and the other is justice in appointment”(FH19).*

### 3.3. Facilitating Transformational Changes

Nursing managers stated that in the healthcare system, especially nursing, there should be a basis for accepting changes, and the changes should be accelerated and facilitated.

To achieve this goal, they consider three key strategies, including motivational communications, building trust, and expanding influence.

#### 3.3.1. Motivational Communications

Motivational communications are one of the most critical strategies for accepting, accelerating, and facilitating changes, which are repeated many times in the experiences of nursing managers. Motivational communications can help nurses be more motivated in performing their professional duties and experience improved performance and job satisfaction. At the same time, this motivation can enhance nurses' communication and have a positive effect on transformations.

Nursing managers use various strategies for motivational communications, such as appreciating the performance of staff, maintaining the dignity of personnel and patients, and providing companionship in problems and cooperation in practice.

Appreciating the performance of personnel can facilitate the improvement of the nursing team's performance and increase the motivation of the nurses to perform their duties better.

*“I understand that each of the personnel does an important job; I appreciate him/her at the first opportunity to motivate him/her…” (FH20)…. “In communications, I appreciate their performance. For example, in the presence of everyone, I encouraged one of the nurses who performed well in the follow-up of patients to motivate others” (MS9).*

Companionship in problems and cooperation in the practice by creating a common atmosphere and positive interactions provides the right grounds for motivating communication.

*“One of the ways I motivate my personnel is by helping them with any problems they encounter. By demonstrating that I stand by them, even during difficult times, I can motivate them to work harder and support me in achieving our goals” (FH23).*

*“In all communications with staff and patients, I maintain their dignity and have never disrespected them”(MS16).*

*“All people have their own personality, and I consider it necessary to never be disrespectful in my behavior to avoid offending patients and staff” (MH21).*

#### 3.3.2. Trust Building

Gaining the trust and confidence of personnel is a critical strategy to accelerate and facilitate change. Nursing managers mentioned the strategies of maintaining working respect and mutual perception, transparency in interactions and feedback, transparency of processes, and consultation strategy.

Maintaining working respect and understanding personnel during problems helps to strengthen human relations and create a healthy environment. Respecting the rights and dignity of staff and maintaining respect in daily dealings improve human relations, and create a mutually respectful environment, and then gain trust and facilitate transformations.

*“In communications, I make sure to behave with all staff with respect, irrespective of their level or position” (MH21). “I have understood my employees during their problems, and I did not leave them alone. That is the reason they trust me….” (MS13).*

Nursing managers believe that transparency in providing honest information, improving interactions and feedback, publishing reports, and announcing performance, as well as transparency in processes, can increase the level of confidence and trust of staff and patients in healthcare organizations and facilitate and improve transformations in nursing.

*“I transfer the information clearly, correctly, and completely between the members of the treatment team and use standard reporting forms (error report form), integrated notification systems (system for registering offers). I encourage nurses to communicate directly and continuously with other team members, and this has been effective in improving information transfer and transparency because I have been able to gain their trust” (FH23).*

Using the experience, knowledge, and capabilities of nurses for clinical and managerial consultation and important decisions can strengthen mutual trust. When personnel collaborate with each other based on consultation, they share each other's opinions and views, and reach the best solutions through joint decision-making and actions. This process strengthens trust and relationships

*“When I seek consult from my staff, they feel valued, and when they see that I trust them, they trust me more” (FMa19).*

#### 3.3.3. Expansion of Influence

Nurses and nursing managers, as key members of the health system, play a vital role in providing care to patients and the community. The relationship between the increase of influence in nursing and the facilitating transformational changes shows that influence is achieved with complete mastery of the managerial field and gaining clinical authority, communication with the nursing practice to accompany the personnel, and role modeling with professional behavior.

Clinical authority allows nursing managers to make more optimal decisions based on their maturity, knowledge, and experience in the nursing practice. They can have the ability to diagnose and make more accurate decisions and choose the best treatment and care options for patients. This can lead to improving the quality of care, improving the health of patients, and their satisfaction.

*“As I gain more experience and become a more robust manager, I earn more tremendous respect from my team. With time and effort, I can bring about meaningful changes in our workplace ”(MS9).*

Accompanying personnel in the transformations to improve the performance of the organization and improve the quality of health care is of great importance. With the active participation of personnel and accompanying them in the organization, it is possible to achieve more influence and successful transformations in nursing.

*“The more they know you at the clinical nursing practice, the more they listen to you and the more they accompany you, and you can solve problems very quickly, and according to the staff, they will do more work and have a more significant impact on the system” (FS22).*

Role modeling and influencing in nursing make nursing managers have the ability to have a greater impact on improving the quality of care, the behavior of patients, and the professional development of their colleagues. By demonstrating professional behavior and effective leadership, nurse managers can serve as inspirational examples for other personnel and create positive changes in the nursing environment and improve patient health.

*“In my behavior, I show respect for the rights of patients, I observe professional ethics in all aspects of work…. I observe discipline…. I have a close and effective relationship with colleagues…. I have cooperation and coordination in care. My best effort is to be an alive role model for my colleagues”(MM24).*

## 4. Discussion

This study explored effective strategies for implementing a transformational approach in nursing practice, identifying three key strategies: drawing the path toward transformation, fostering a transformation-based culture, and facilitating transformational changes.

While previous studies have extensively discussed the transformational approach across various professions, including nursing [23], this study adds to the growing body of research by highlighting the specific strategies that nursing managers employ to drive transformational change. The findings align with the study of Sunmi Kim, which indicated that nursing managers adopting transformational leadership practices can improve empowerment, nursing performance, job satisfaction, and organizational commitment among nursing staff [24]. Similarly, Phinari found that transformational practices positively impact nurses' performance and motivation [12]. These studies underscore the importance of transformational leadership in nursing practice, echoing the results of our study.

Regarding the first category of the present study, “Drawing the Path of Transformation”: This category explores the strategies nursing managers use to establish a clear and effective pathway for transformation within their teams. It includes the recognition of transformations, goal setting based on transformation and creativity in the path of transformation. The strategies associated with the transformational approach aim to drive change by fostering a culture of innovation and problem-solving among staff. As Weng noted, the innovative behavior of individuals or groups is heavily influenced by the organizational climate, with transformational leadership acting as a key driver by shaping this climate [25]. Our findings are consistent with this perspective, as nursing managers emphasized creativity as a cornerstone of transformation. They advocated for brainstorming sessions and the exploration of novel ideas to cultivate innovation within nursing practice.

The study's findings resonate with those of Massen who emphasized the need for a positive work environment within healthcare settings to foster mutual respect, trust, and open communication among staff [26]. Our findings also support Massen's conclusions, as strategies such as creating a motivating atmosphere, strengthening support, and fostering creativity were common between the two studies.

Despite generally positive attitudes toward innovation among nurses, access to evidence-based information and resources often remains inadequate [27]. Proposing and implementing innovations in nursing science leads to important outcomes such as the acquisition of new knowledge and skills, enhanced scientific recognition for nurses, the creation of a distinct work culture, and improved financial results. Nurse leaders are pivotal in cultivating a culture of innovation, motivating nurses to engage in ongoing education, supporting the development of innovative practices, and taking an active role in these initiatives [28]. To capitalize on these benefits, healthcare organizations should prioritize educational initiatives, including seminars and training programs on innovation, while also ensuring access to evidence-based resources [27].

Regarding the second category of the present study, “Fostering a Transformation-Based Culture”: This category examines how nursing managers cultivate a culture that supports transformational change. This involves the dissemination of transformative thinking and a different perspective on the organization's capital. One key contribution of this study is the emphasis on fostering a transformation-oriented culture, where the dissemination of transformational thinking and a shift in perspective toward staff and organizational resources are crucial. This aligns with broader studies on the transformational approach, which suggests that leadership focused on building relationships and engaging with staff enhances organizational effectiveness and improves patient outcomes [29]. Effective interpersonal relationships as a strategy promote learning and lead to a more complete understanding of people [30]. Furthermore, Eline de Kok advocates for organizational strategies that encourage nurses to exhibit leadership abilities within a positive work setting, aligning with our emphasis on building trust and fostering a culture based on transformation [31].

Cultural values also play a significant role in organizational effectiveness, as Doody emphasized. He linked effectiveness to the interaction between organizational goals and results while highlighting the importance of recognizing and valuing staff contributions. Doody also underscored the need for future nurse leaders to foster creativity and passion among their teams to address challenges and achieve meaningful transformations in healthcare services [32]. These insights align with our findings, which stress the importance of guiding staff in alignment with organizational values and fostering an environment that encourages creativity and challenges the status quo.

Recent studies have shed light on the role of organizational capital in nursing. Psychological and human capital development positively influence nurses' work engagement and organizational citizenship behaviors [33]. Notably, work engagement serves as a mediator between psychological capital and organizational citizenship behaviors, presenting opportunities to enhance nursing performance through targeted strategies [34].

Nurse managers are responsible for effectively utilizing both the human capital and social capital within their teams to ensure the achievement of high-quality outcomes. However, the relationship between these two forms of capital and their combined influence on organizational outcomes through nursing leadership remains underexplored. The Gilbert Conceptual Model of Organizational Intellectual Capital underscores the critical role of nurse managers in optimizing these resources to enhance team and organizational performance [35]. This model serves as a valuable framework for advancing research on the impact of nursing leadership on the development and utilization of organizational intellectual capital. In our study, we identified that improving relationships with staff and involving them in decision-making were key strategies for fostering a transformation-driven culture.

Regarding the third category of the present study, “Facilitating Transformational Changes”: This category focuses on the practical steps nursing managers take to facilitate and manage the changes required for transformational leadership. It includes strategies such as motivational communications, building trust, and expanding influence.

The transformational approach remains a critical framework for individuals navigating environmental changes, particularly in dynamic work settings, as it enhances organizational commitment and job satisfaction [29]. Cultural values and norms significantly influence leadership styles, shaping how transformational leadership is applied and received by nursing staff. In regions like Asia, factors such as hierarchy, communication styles, and power distance impact leadership effectiveness. While the study identifies the importance of leadership practices such as communication, trust building, and expanding influence, there is a need for a more nuanced understanding of how these strategies are applied across different cultural contexts. By understanding these cultural nuances, nursing managers can adapt their strategies to ensure they are both universally applicable and contextually relevant, improving their effectiveness in diverse healthcare settings.

Skilled nurse managers employ diverse power bases, influence strategies, and conflict resolution techniques to guide their teams effectively [36]. Clinical authority in nursing emerges as a critical factor in enhancing healthcare service quality. Its implementation has been shown to improve nurse performance by fostering accountability, adherence to standards, and better communication [37]. However, the extent of its application varies across healthcare settings and nursing roles. While some institutions have adopted assessment systems grounded in clinical authority since 2015 [38], others are still in the process of achieving full implementation [39]. To optimize outcomes, aligning clinical authority with nurses' career progression and competencies is imperative [37]. These results are consistent with our study.

However, this study's generalizability is limited due to its qualitative nature and the variability in nursing managers' strategies across different cultural and contextual settings. Further research employing diverse methodologies is needed to clarify the strategies required for a transformational approach in varied contexts. Additionally, the study's focus on experienced nursing managers—with a mean of 18 years of professional experience and 11 years of management—may have skewed the findings toward strategies more commonly used by seasoned professionals.

## 5. Conclusion

This study highlights the transformational strategies employed by nursing managers, focusing on establishing clear pathways for transformation, fostering a culture of change, and acting as facilitators throughout the transformational process. The study revealed that these strategies are integral to promoting innovation, adaptability, change, and a transformation-oriented culture. By consistently implementing and refining these strategies, nursing managers can significantly enhance organizational effectiveness, improve patient outcomes, and drive meaningful advancements in nursing practice. The active support and acceleration of change through these strategies not only strengthens a culture centered around transformation but also fosters a work environment that encourages continuous learning and improvement.

Future studies could explore the implementation of transformational leadership strategies across various healthcare settings and cultures to identify best practices and challenges. Additionally, examining the influence of nursing managers' personal leadership styles on the effectiveness of these strategies, as well as investigating nursing staff perceptions of transformational leadership, could provide valuable insights into its impact on team dynamics and job satisfaction.

## Figures and Tables

**Table 1:** Interview questions.

General question	Please share your experiences of a typical working day?

Middle question	What are the objectives you seek to achieve through your working day?
	What strategies do you follow to advance the goals in the organization?
	Can you elaborate on that?
	What does that mean?

Final question	Are there any additional keywords, ideas, or statements you believe were missed during the interview?

**Table 2:** Demographic characteristics of the participants.

Participant/current position	Gender	Experience (years)	Management experience (years)	Education
P1. Supervisor	F	23	18	MSc
P2. Head nurse	M	18	7	BSN
P3. Supervisor	F	17	10	MSc
P4. Head nurse	F	19	16	BSN
P5. Head nurse	M	17	15	BSN
P6. Head nurse	F	20	8	MSc
P7. Supervisor	M	16	4	MSc
P8. Hospital matron	M	24	14	PhD
P9. Supervisor	F	18	11	MSc
P10. Head nurse	M	21	19	BSN
P11. Head nurse	F	15	4	BSN
P12. Supervisor	F	22	9	MSc
P13. Head nurse	F	23	16	MSc
P14. Head nurse	M	18	7	BSN
P15. Supervisor	M	17	17	MSc
P16. Head nurse	F	19	15	BSN
P17. Head nurse	M	25	19	BSN
P18. Head nurse	F	18	18	MSc
P19. Hospital matron	F	19	10	PhD
P20. Supervisor	M	13	5	MSc
P21. Hospital matron	M	11	3	MSc
P22. Supervisor	M	9	6	MSc
P23. Supervisor	M	12	5	MSc

Abbreviations: BSN, Bachelor of Science in Nursing; F, Female; M, Male; MSc, Master of Science; PhD, Doctor of Philosophy.

## Data Availability

The data that support the findings of this study are available from the corresponding author upon reasonable request.
